# Food for Dyspeptics

**Published:** 1891-12

**Authors:** 


### FOOD FOR DYSPEPTICS.

I wish to tell the sufferer from a weak stomach how to cook some things which a dyspeptic can eat. Four years ago my husband was almost helpless with dyspepsia. He consulted two doctors from whom he learned that he could 'not live a year.' Every thing he ate caused great pain, until he tried a fresh egg, well beaten with a little sugar, a very little salt and nutmeg, over which was poured a teacup two-thirds full of boiling milk, stirring the egg constantly. He took this warm and could retain it without trouble. Later I prepared milk toast for him as follows: I used stale, salt-rising bread, made from wheat middlings, cut in slices half an inch thick, toasted a nice brown in a brisk oven and soaked in sweet milk which had been boiled and slightly thickened with flour and seasoned with salt and butter. Another dish consisted of one cup of rice, well washed, put in a large granite basin with one cup of water and half a teaspoon of salt, and allowed to cook slowly until all the water was taken up in the rice. Then I added two tablespoonfuls of sugar, and five cupfuls of new milk and stirred it well, after which I baked it in a slow oven for several hours. The rest of the family liked this as well as he did, especially when served with sweet sauce.

This is the way I made dyspeptic corn-cake. I took one egg, one tablespoonful of brown sugar, one-half teaspoonful of salt, one-half a pint of sour cream, one pint of sour buttermilk, three-fourths of a teaspoonful of soda and one teaspoonful of baking powder. I beat the egg and sugar together until very light, stir in the cream and salt, then the buttermilk, next the soda dissolved in a little warm water, and make all into a stiff batter with three parts corn-meal to two parts of fine flour into which the baking powder has been sifted. I set the dish in the steamer, let it steam three hours, then bake it twenty minutes in a hot oven.—M. L. D.

